# SumoPred-PLM: human SUMOylation and SUMO2/3 sites Prediction using Pre-trained Protein Language Model

**DOI:** 10.1093/nargab/lqae011

**Published:** 2024-02-07

**Authors:** Andrew Vargas Palacios, Pujan Acharya, Anthony Stephen Peidl, Moriah Rene Beck, Eduardo Blanco, Avdesh Mishra, Tasneem Bawa-Khalfe, Subash Chandra Pakhrin

**Affiliations:** Department of Computer Science and Engineering Technology, University of Houston-Downtown, 1 Main St., Houston, TX 77002, USA; Department of Computer Science and Engineering Technology, University of Houston-Downtown, 1 Main St., Houston, TX 77002, USA; Department of Biology and Biochemistry, Center for Nuclear Receptors & Cell Signaling, University of Houston, Houston, TX 77204, USA; Department of Chemistry and Biochemistry, Wichita State University, 1845 Fairmount St., Wichita, KS 67260, USA; Department of Computer Science, University of Arizona, 1040 4th St., Tucson, AZ 85721, USA; Department of Electrical Engineering and Computer Science, Texas A&M University-Kingsville, Kingsville, TX 78363, USA; Department of Biology and Biochemistry, Center for Nuclear Receptors & Cell Signaling, University of Houston, Houston, TX 77204, USA; Department of Computer Science and Engineering Technology, University of Houston-Downtown, 1 Main St., Houston, TX 77002, USA

## Abstract

SUMOylation is an essential post-translational modification system with the ability to regulate nearly all aspects of cellular physiology. Three major paralogues SUMO1, SUMO2 and SUMO3 form a covalent bond between the small ubiquitin-like modifier with lysine residues at consensus sites in protein substrates. Biochemical studies continue to identify unique biological functions for protein targets conjugated to SUMO1 versus the highly homologous SUMO2 and SUMO3 paralogues. Yet, the field has failed to harness contemporary AI approaches including pre-trained protein language models to fully expand and/or recognize the SUMOylated proteome. Herein, we present a novel, deep learning-based approach called SumoPred-PLM for human SUMOylation prediction with sensitivity, specificity, Matthew's correlation coefficient, and accuracy of 74.64%, 73.36%, 0.48% and 74.00%, respectively, on the CPLM 4.0 independent test dataset. In addition, this novel platform uses contextualized embeddings obtained from a pre-trained protein language model, ProtT5-XL-UniRef50 to identify SUMO2/3-specific conjugation sites. The results demonstrate that SumoPred-PLM is a powerful and unique computational tool to predict SUMOylation sites in proteins and accelerate discovery.

## Introduction

Post-translational modifications (PTMs) are the predominant factors leading to the diversity of the proteome (1,2). Protein SUMOylation is one of the most common PTMs in humans that performs essential roles in many vital biological processes like transcription control, chromatin organization, accumulation of macromolecules in cells, regulation of gene expression, and signal transduction (3,4). SUMOylation is also necessary for the conservation of genome integrity (5). Consequently, it is not surprising that a change in SUMOylation dynamics favors the onset of a variety of human diseases including cancer, Alzheimer's disease, Parkinson's disease, viral infections, heart diseases, and diabetes (5–9).

SUMOylation occurs as a modifier in an ϵ-amino group of lysine residues in the target protein through a multi-enzymatic cascade (10). In this reaction, SUMO is connected to a lysine residue in substrate protein by covalent linkage via three enzymes, namely activating (E1), conjugating (E2) and ligase (E3). Also, it can be separated from the target protein by a specific SUMO protease enzyme (11). This covalent SUMO conjugation frequently occurs at a consensus motif $\psi$-K-X-E where $\psi$ represents lysine, isoleucine, valine, or phenylalanine, K is lysine, X can be any amino acid, and E is glutamic acid (12). However, additional SUMO protein substrates have recently been identified that lack this canonical SUMO consensus motif, making it more challenging to identify SUMOylated protein targets.

SUMOylation requires the Small Ubiquitin-Related Modifier (SUMO) protein, which has structural similarity to ubiquitin and has been discovered in a wide range of eukaryotic organisms (6,13,14). Five SUMO paralogues exist in humans with SUMO1, SUMO2 and SUMO3 expressed ubiquitously in multiple tissue/cell types and consistently the most studied. SUMO2 and SUMO3 are highly homologous with 97% amino acid sequence overlap and frequently referred to as SUMO2/3. Unlike SUMO1, SUMO2/3 includes an internal SUMO-consensus site with lysine 11, which allows for poly-SUMOylation to occur (15). The internal SUMOylation site allows SUMO2/3 to form poly-SUMO chains on target proteins. The SUMO2/3 poly-chain serves as a binding platform for proteins with SUMO-interaction motifs (SIMs) and thereby supports dynamic non-covalent protein complexes. As SUMO2/3 directs both covalent and non-covalent protein interactions, previous biochemical studies suggest a unique protein substrate profile and biological function for SUMO2/3 versus SUMO1. SUMO2/3 specific protein conjugates direct protein degradation, chromatin remodeling, gene expression, and DNA repair (9,16,17). Also, only the SUMO2 knockout is embryonic lethal and is essential for organismal development (18,19). Yet identification of SUMO paralogue specific targets is still in its infancy and is frequently only addressed at an individual protein level.

To date, SUMOylation is most often identified using mass spectrometry and a lot of progress has been made in experimental techniques used for mapping and quantifying PTMs. In that regard, more than 53 000 unique SUMOylation sites have been identified in human proteins (20–22). Although experimental approaches are the most reliable ways to identify SUMOylation sites, they are often time-consuming, labour-intensive, and are still quite limited. Thus, a mechanistic characterization of PTMs including SUMOylation sites is lacking for a large portion of the proteome. Therefore, complementary computational tools using machine learning (ML) and deep learning (DL) are playing an increasingly essential role in the characterization of SUMOylation sites.

Several different SUMOylating site prediction models currently exist (4,23–35). The most utilized program remains GPS-SUMO with the ability to predict SUMO-accepting lysine residues in consensus and non-consensus SUMO sites (36). However, like most prediction models, the input features are still hand-crafted features. Additionally, to the best of our knowledge the benefits of the recent advances in large protein language models (PLMs) and the distributed representation learned from the distillation of these language models have not been explored for SUMOylation site prediction. A recent study evaluated the performance of different models for protein representation, revealing that ProtT5 achieved the best performance in most of the tasks (37). However, ProtT5 has not yet been used for SUMOylation and SUMO2/3 PTM prediction.

Recently, transformer-based language models trained with a large corpus of unlabelled data have achieved stunning results in the field of natural language processing (NLP) (38). Due to the availability of large number of protein sequences in the UniProt knowledge base and other resources, we now have a wide variety of PLMs under development (39–46). Considering protein sequences as sentences, Elnaggar *et al.* developed a pre-trained PLM called ProtT5-XL-UniRef50 (herein called ProtT5) based on 2.5 billion protein sequences (46). The representations of these models have been utilized for various downstream tasks, and the results demonstrate that the distributed representation learned from the distillation of these language models provide useful information that captures the evolutionary context of a sequence, contact map, taxonomy, long-range dependencies, protein structure, physicochemical properties, subcellular localization, and function (47–53). Moreover, long-range dependencies can yield essential insights into the broader context and functional implications of SUMOylation PTM. These dependencies offer indispensable insights into the intricate connections between distant amino acids within a protein, shedding light on how modifications at one site influence the protein's behavior and its interactions with other molecules. Taking these distant relationships into account can enhance the accuracy of algorithms used to predict SUMOylation PTM sites. Additionally, it can also provide valuable information about the protein's three-dimensional structure. For example, it can help pinpoint regions of a protein that, although far apart in the primary sequence, exhibit close interactions within the folded protein structure, which is pertinent for predicting SUMOylation PTM sites. Similarly, features from these transformer-based PLMs have been successfully utilized to predict signal peptides (54), lysine glycation sites (55), *N* and O-linked glycosylation sites (51,56), phosphorylation sites (57), lysine crotonylation sites (58), subcellular localization (59), protein structural features (60), intrinsic disorder sites (61), and binding residues (52) among others.

Hence, we propose a novel computational approach called SumoPred-PLM (**S**UMOylation site **P**rediction using **P**rotein **L**anguage **M**odel) that utilizes embeddings from a protein language model (i.e. ProtT5) to improve the predictive performance of SUMOylation sites. By considering proteins as sentences, we feed the full protein sequence into the pre-trained ProtT5 model to extract fixed-length high-dimensional per residue representations from the last encoder layer. Subsequently, the high-dimensional contextualized embeddings (i.e. a vector with 1024 features) of the site in interrogation (Lysine, K) are fed into a Deep Neural Network (DNN), essentially a Multi-layer Perceptron (MLP)-based classifier for SUMOylation and SUMO2/3 site prediction.

Using cross-validation experiments, we found that the classifier based on the MLP architecture performed better compared to other architectures employed. To demonstrate its effectiveness, we evaluated the performance of the proposed method SumoPred-PLM using the GPS-SUMO dataset against quintessential approaches like GPS-SUMO (36). Our experiments showed that SumoPred-PLM achieved better performance in predicting protein SUMOylation sites compared to the state-of-the-art GPS-SUMO predictor, yielding an area under the receiver operating characteristic curve (AUROC) of 0.895. SumoPred-PLM is a freely available, fast and reliable approach for prediction of SUMOylation sites. All programs and data are available at https://github.com/PakhrinLab/SumoPred-PLM.

## Materials and methods

### Predicting protein SUMOylation and SUMO2/3 sites

This section describes the dataset, features extraction method, performance evaluation metrics, feature selection, and the methods for training the model. With the aim to train a DL algorithm to predict SUMOylation and SUMO2/3 sites in proteins, we utilized three different datasets: CPLM 4.0 (22), SUMO2/3 (62) and GPS-SUMO (36), which are described below.

### CPLM 4.0 dataset

In this study we utilized the Compendium of Protein Lysine Modifications (CPLM 4.0) dataset that was developed by Zhang *et al.* (22). This dataset consists of 29 different types of lysine PTMs including SUMOylation as a part of the CPLM 4.0 database. To avoid overestimation of the prediction accuracy as well as to maintain diversity, the redundant sites were removed using CD-HIT Suite with a threshold of 30% sequence identity (63). If two or more proteins were found to be modified at the same position and if they have >30% sequence similarity, only one of the proteins was preserved. As a result of this filtering, we obtained 5,695 unique and diverse SUMOylated proteins. Moreover, we separated 5117 SUMOylated proteins for the training. From these training proteins we extracted 26 911 positive samples (SUMOylated sites) and 201 949 negative samples (non-SUMOylate sites). Additionally, we extracted 2988 independent positive and 2988 independents negative SUMOylated sites distributed across 578 proteins for testing. SUMOylation may occur on any lysine (K) amino acids of the SUMOylated protein sequence; however, not all of these sites are SUMOylation sites. We considered the experimentally verified SUMOylated sites acquired from the CPLM 4.0 database as positive SUMOylated sites. All other lysine sites within the same substrate are considered as negative SUMOylation sites. The difference between the small number of positive and large number of negative samples makes this benchmark dataset unbalanced. This imbalance can bias the performance of any predictor towards the identification of negative samples (a high true negative rate) over the detection of positive samples (a low true positive rate).

The two commonly used strategies to overcome the imbalance problem are random over-sampling and under-sampling. The idea behind over-sampling is to duplicate the positive samples to increase them to the number of negative samples. While in under-sampling, some of the negative samples are discarded to make the number of negative samples equal to the number of positive samples. The over-sampling procedure could increase the probability of over-fitting the model due to duplication of positive samples while under-sampling often provides a modest solution for a given model. Therefore, we selected an under-sampling procedure to overcome the imbalance problem (64). As a result of under-sampling, we ended up with 26,911 positive and an equal number of negative samples. In this way, we avoid bias in our benchmark towards negative samples and increase our chance to detect more positive samples, or in other words, more SUMOylation sites accurately. Table 1 shows the number of positive and negative sites from CPLM 4.0 Dataset after 30% CD-HIT. Moreover, we explored the dbPTM human SUMOylation dataset (65). Supplementary Table S1 presents the statistics of the training and independent test datasets when 30% psi-cd-hit was applied.

**Table 1.** Positive and negative SUMOylation sites for training and independent testing derived from CPLM 4.0 dataset

Dataset	Number of Proteins	Positive	Negative
**Training**	5117	26 911	201 949
**Test**	578	2988	2988

### SUMO2/3 dataset

Another dataset we utilized in this study is the human endogenous SUMO2/3 SUMOylation dataset developed by Hendriks *et al.* (62). This dataset consists of 14 869 endogenous SUMO2/3 Sites. We used CD-HIT Suite with a threshold of 30% to remove sequence identity among SUMO2/3 proteins (63). As a result of this filtering, we obtained 3225 SUMOylated proteins. Moreover, we separated 2902 SUMOylated proteins for training. From these training proteins we extracted 10 684 positive sites (SUMO2/3 sites) and 10 684 randomly under-sampled negative sites (non-SUMO2/3 sites) from 131 459 negative sites. Additionally, we extracted 1269 independent positive and 1269 independents negative SUMO2/3 sites distributed across 322 proteins for testing. We considered the experimentally verified SUMO2/3 sites acquired from Hendriks *et al.* database as positive SUMO2/3 sites and all the other lysine sites within the same substrate as negative SUMO2/3 sites. Table 2 shows the number of positive and negative sites from Hendriks *et al.* SUMO2/3 dataset after 30% CD-HIT.

**Table 2.** Positive and negative SUMO2/3 sites for training and independent testing derived from Hendriks *et al.* dataset

Dataset	Number of proteins	Positive	Negative
**Training**	2902	10 684	131 459
**Test**	322	1269	1269

### GPS-SUMO dataset

The GPS-SUMO dataset consists of 548 proteins and among them 509 proteins were used for training, and 39 were utilized for independent testing. Eight hundred ninety one experimentally verified human SUMOylation sites were extracted from 509 SUMOylation proteins. All the other lysine's from the same 509 SUMOylated proteins were considered as negative SUMOylation sites. To create a balanced dataset, 891 negative sites were randomly under sampled from 23 371 sites. These experimentally verified sites form the training data. Moreover, 71 experimentally verified independent positive SUMOylated test sites were extracted from 39 different SUMOylation protein which are different than the training proteins. Next, 1377 independent negative lysine sites which do not include the independent SUMOylated positive sites were extracted from the same 39 independent SUMOylation proteins. These experimentally verified sites form the testing dataset. Further information about GPS-SUMO can be found in the seminal approach section of GPS-SUMO (36). Table 3 summarizes the number of sites included in GPS-SUMO dataset.

**Table 3.** Positive and negative SUMOylation sites for training and independent testing derived from the GPS-SUMO dataset

Dataset	Number of Proteins	Positive	Negative
**Training**	509	891	23 371
**Test**	39	71	1377

### Feature extraction—embeddings from protein language model

A range of numerical representation schemes can be used to encode protein sequences. A recent development in the field is the advent of embeddings (distributed vector representations), which are representations of protein sequences extracted from the last hidden layers of the networks forming the PLM trained on a large set of unlabeled protein sequences. These latent embeddings capture a diversity of higher-level features of proteins and have been used successfully in predicting secondary structure and other tasks (52). In this work, we used embeddings from the PLM, ProtT5-XL-Uniref (herein called, ProtT5) (46). The PLM ProtT5 was trained on unlabeled protein sequences from BFD (Big Fantastic Database; 2.5 billion sequences including meta-genomic sequences) (66), and UniRef50 (67). ProtT5 has been built in analogy to the NLP (Natural Language Processing) T5, ultimately learning some of the constraints of protein sequences (68). Features learned by the PLM can be transferred to any (prediction) task requiring numerical protein representations by extracting vector representations for single residues from the hidden states of the PLM using transfer learning. As ProtT5 was only trained on unlabeled protein sequences, there is no risk of information leakage or overfitting to a certain level during pretraining. Essentially, ProtT5 outputs fixed length (1024) vector representations for each residue in a protein sequence. In essence, to predict whether an amino acid lysine is SUMOylated, SUMO2/3 or not, we extracted a 1024-dimensional vector for each SUMOylated, SUMO2/3 or non-SUMOylated, non-SUMO2/3 lysine residue, where only the encoder side of ProtT5 was used, and embeddings were extracted from the last hidden layer of the models. A similar methodology was applied to extract features from influential Ankh PLM (41). We utilized the Ankh large model because our experimental results show that it can encode more intrinsic information about proteins than the Ankh base model. The only difference from the ProtT5 model was that it produced a per residue contextualized embedding feature vector length of 1536 rather than 1024 produced by ProtT5.

### Machine learning and deep learning models

Naïve Bayes (NB) is a simple ML algorithm commonly used for classification tasks (69). It is based on Bayes theorem and assumes that the features are conditionally independent given the class label.

Support Vector Machine (SVM) is a class of supervised machine learning algorithms used for classification and regression tasks (70). The basic idea behind SVM is to find an optimal hyperplane that separates the data into different classes. When the data is not linearly separable, SVM can still classify it by using kernel trick. The kernel trick maps the input data into a higher-dimensional feature space, where it might become linearly separable.

Random Forest (RF) is a popular ensemble learning method used for classification and regression tasks in ML (71). It is an extension of decision trees and combines multiple decision trees to make predictions. For classification tasks, it predicts the class label by taking a majority vote among the individual trees. Each tree's prediction is counted, and the class with the most votes becomes the final prediction.

Logistic Regression (LR) is a ML algorithm used for binary classification tasks (72). It predicts the probability of an instance belonging to a certain class by fitting a logistic (sigmoid) function to the input features. It estimates coefficients to create a linear decision boundary that separates the two classes.

Extreme Gradient Boosting (XGBoost) belongs to the family of gradient boosting method (73). It sequentially adds weak models (decision trees) to iteratively correct the errors made by previous models. It optimizes a specific loss function by finding the best-fitting model in an additive manner.

1D Convolutional Neural Network (1D CNN) is a variant of convolutional neural networks (CNNs) specifically designed for processing one-dimensional sequential data (74). It utilizes one-dimensional convolutional filters to capture local patterns and features in sequential data. The filters slide along the input sequence, performing convolutions and generating feature maps. While traditional CNNs are commonly used for image analysis and computer vision, 1D CNN is particularly suited for tasks involving sequential data, such as time series analysis, speech recognition, and natural language processing. Long Short-Term Memory (LSTM) is a type of recurrent network (RNN) architecture specifically designed to model and process sequential data. It addresses the vanishing gradient problem that occurs in traditional RNNs, allowing for better capturing of long-term dependencies in the data. The hyperparameters and other details are explained in Supplementary Table S2.

### Model training

**Figure 1.**
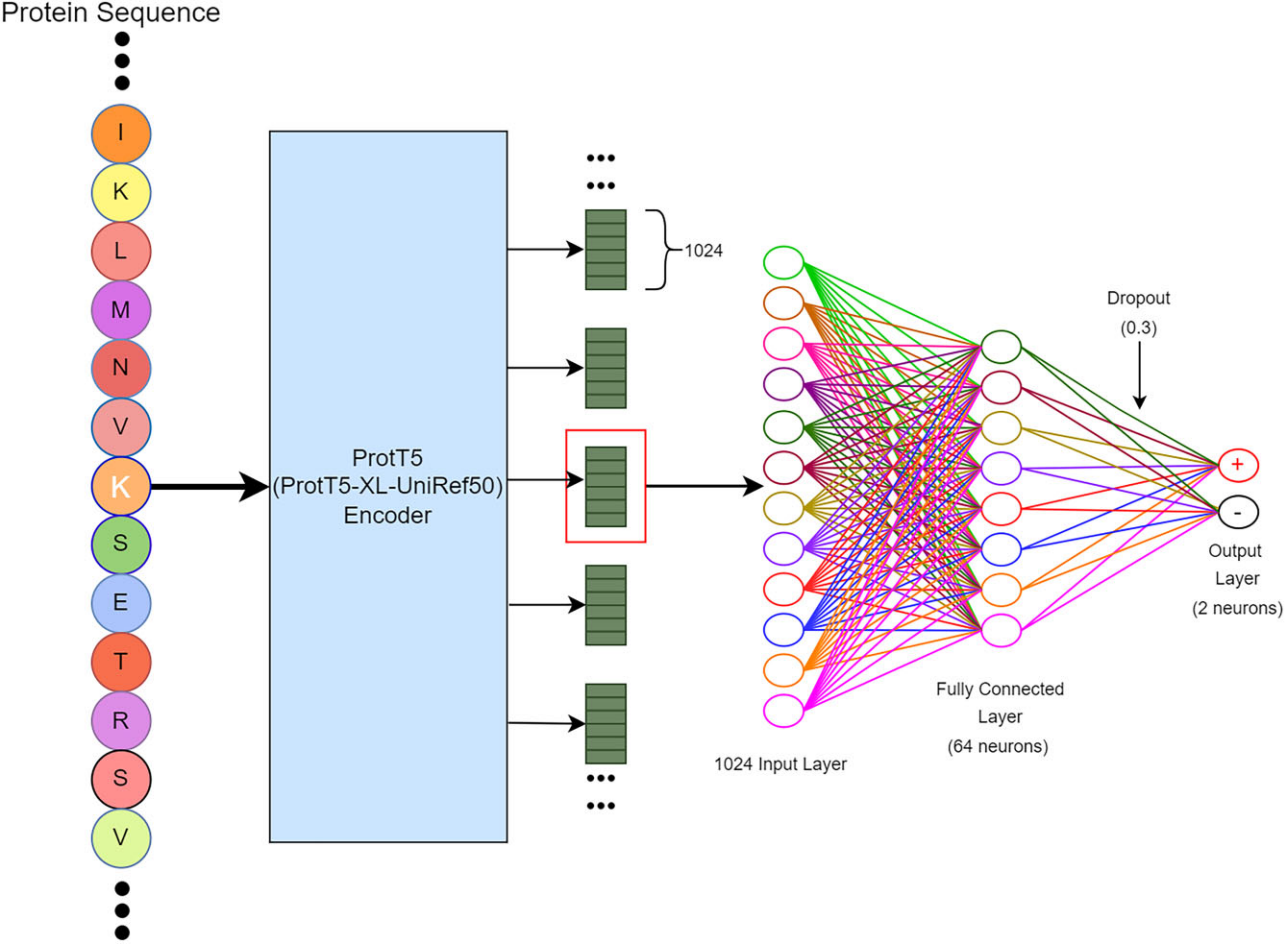
The overall framework of SumoPred-PLM. Beads with letters represent protein sequences. The sky-colored rectangular box represents ProtT5 PLM. Green rectangular boxes are per residue 1024 features representations produced by ProtT5 PLM. The empty circle represents neurons. Each neuron is connected to other nodes via links like a biological axon-synapse-dendrite connection. A dropout of 0.3 means, 30% of neurons are switched off randomly while training the MLP.

As discussed above, SUMOylation and SUMO2/3 occur on lysine residues, so we extract contextualized embeddings from the ProtT5 model using the full-length protein sequence as input. Finally, the corresponding feature for the site of interrogation (in this case lysine) is extracted (1024-dimensional vector) and passed to the subsequent DL model. Using these representations and datasets (CPLM 4.0, SUMO2/3, and GPS-SUMO), we trained several models to correctly predict SUMOylation, and SUMO2/3 sites in amino acid sequences. The performance of several architectures was evaluated: 1D CNN, 1D CNN-LSTM, 1D CNN-BiLSTM, BiLSTM, LSTM, LR, MLP, SVM, XGBoost, NB and RF. We describe the MLP architecture in Figure 1. As shown in Figure 1, the features are extracted for the site of interrogation (K, highlighted in white) using full protein sequence as input and the 1024 real-valued feature vectors are fed into a MLP deep-learning architecture consisting of 64 neuron input layers followed by 2 neuron output layers. To explore the hyperparameter space, we performed a ten-fold cross-validation grid search on the MLP deep learning model with the CPLM 4.0, and SUMO2/3 training dataset. It was done against 1, 2, 3 and 4 dense hidden layers; sigmoid and ReLU activation function; 32, 64, 128, 256, 512 and 1024 neurons in each layer; RMSprop, and Adam optimizers; and 0.2, 0.3, 0.4 and 0.5 dropout rate; whereas the default learning rate of 0.001 was used. A similar approach was performed for the rest of the deep learning and machine learning algorithms. The optimized hyperparameters using grid search are shown in Table 4. Based upon grid search, 64 neuron input layers were configured with ReLU activation function. As dropout layer/nodes in the network helped alleviate overfitting and improved the generalization capacity, we set the dropout equal to 0.3. Our task was to train a binary classification model to distinguish SUMOylation or SUMO2/3, and non-SUMOylation or SUMO2/3 sites. Therefore, in the output dense layer, we set the number of neurons equal to 2. The optimized hyperparameters for the deep-learning architecture are elaborated in Table 4. To avoid overfitting, we have used overfitting reduction techniques like dropout, early stopping, model checkpoint, and reduce learning rate on the plateau. Furthermore, no signs of overfitting and underfitting are present in our trained model as can be seen from Supplementary Figure S1. The loss curve for the training and validation follows each other as well as the training and validation accuracy curves also follow each other.

**Table 4.** Hyperparameters used in the MLP network for the SUMOylation, SUMO2/3 and GPS-SUMO datasets

Name of the Parameters	Value Used
No. of layers	1
No. neuron in dense layers	64
No. of neuron in the output layer	2
Activation Function	ReLU
Activation Function at output layer	SoftMax
Optimizer	Adam
Learning rate	0.001
Objective / loss function	Binary cross entropy
Model Checkpoint	Monitor = ‘Validation accuracy’
Reduce learning rate on plateau	Factor = 0.001
Early stopping	Patience = 5
Dropout	0.3
Decision Boundary	0.5
Batch size	256
Epochs	400

### Model evaluation and performance metrics

In this study, 10-fold cross-validation was used to evaluate the performance of the model and to determine its robustness and generalizability. During 10-fold cross-validation, the data are partitioned into ten equal parts. Then, one part is left out for validation while training is performed on the remaining nine parts. This process is repeated until all parts are used for validation. For the results of 10-fold cross-validation, unless otherwise noted, all performance metrics are reported as the mean value ± 1 standard deviation from the mean.

To evaluate the performance of each model, we use accuracy (ACC), sensitivity (SN), specificity (SP) and Matthews correlation coefficient (MCC) (75,76). ACC describes the correctly predicted residues out of the total residues (Equation (1)). Meanwhile, SN defines the model's ability to distinguish positive residues (Equation (2)), and SP measures the model's ability to correctly identify the negative residues (Equation (3)). On the other hand, MCC considers the model's predictive capability concerning both positive and negative residues (Equation (4)).


(1) \begin{eqnarray*}Accuracy = \;\frac{{TP + TN}}{{TP + TN + FP + TN}}\; \times 100\end{eqnarray*}

(2) \begin{eqnarray*}Sensitivity = \;\frac{{TP}}{{TP + FN}}\; \times 100\end{eqnarray*}

(3) \begin{eqnarray*}Specificity = \;\frac{{TN}}{{TN + FP}}\; \times 100\end{eqnarray*}

(4) \begin{eqnarray*}MCC = \;\frac{{\left( {TP} \right)\left( {TN} \right) - \left( {FP} \right)\left( {FN} \right)}}{{\sqrt {\left( {TP + FP} \right)\left( {TP + FN} \right)\left( {TN + FP} \right)\left( {TN + FN} \right)} }} \nonumber\\ \end{eqnarray*}

## Results

SumoPred-PLM utilizes per residue embeddings (1024 features) extracted for the site of interest (K) from ProtT5 using a full-length sequence as input. We use three datasets for training SumoPred-PLM: CPLM 4.0, SUMO2/3, and GPS-SUMO. Protein redundancies are removed from within and across training and independent test datasets. We performed 10-fold cross-validation on the training dataset(s) to obtain the best hyperparameters for our deep learning architecture. Finally, we used the hyperparameters obtained from 10-fold cross-validation and trained the model using the overall training set and assessed the trained model on the independent test set and compared the performance against other existing approaches.

### Performance on the CPLM 4.0 dataset

#### 10-fold cross-validation on the CPLM 4.0 training set with ProtT5 features

To tune the hyperparameters (parameters whose values are used to control the learning process) and to investigate the performance of various DL/ML models, we performed 10-fold cross-validation on the CPLM 4.0 training dataset (77). The predictive performance of different DL and ML models using the stratified 10-fold cross-validation on the CPLM 4.0 training data set is shown in Table 5. The contextualized embedding of the SUMOylated or non- SUMOylated token ‘K’ produced by the pretrained ProtT5 model when fed to MLP achieves the best performance as seen in Table 5. Intriguingly, the same architecture (MLP) produced the highest result using 10-fold cross-validation on the SUMO2/3 training data set as well. This MLP model produced MCC, SN, SP, and ACC values of 0.478 ± 0.010, 0.757 ± 0.026, 0.720 ± 0.026 and 0.738 ± 0.005 respectively for the stratified 10-fold cross-validation. Since the MLP model produced the best result on 10-fold cross-validation, we selected this architecture as our final model and called it SumoPred-PLM. Furthermore, we conducted a 10-fold cross-validation on the CPLM 4.0 training dataset with the 1D CNN-BiLSTM, 1D CNN-LSTM, BiLSTM and LSTM DL methods. The findings are presented in Supplementary Table S3, revealing subpar performance of these models.

**Table 5.** Results of the 10-fold cross-validation on the CPLM 4.0 training dataset using different deep and machine learning models encoded with ProtT5 PLM. The highest values in each column are highlighted in bold

Models	MCC ± 1 S.D.	SN ± 1 S.D.	SP ± 1 S.D.	ACC ± 1 S.D.
MLP	**0.478 ± 0.010**	**0.757 ± 0.026**	0.720 ± 0.026	**0.738 ± 0.005**
LR	0.444 ± 0.009	0.730 ± 0.006	0.713 ± 0.006	0.722 ± 0.004
XGBoost	0.390 ± 0.011	0.701 ± 0.006	0.689 ± 0.007	0.695 ± 0.005
RF	0.331 ± 0.008	0.578 ± 0.007	0.748 ± 0.005	0.663 ± 0.004
NB	0.229 ± 0.012	0.448 ± 0.006	**0.768 ± 0.007**	0.608 ± 0.005
SVM	0.461 ± 0.022	0.729 ± 0.017	0.732 ± 0.015	0.730 ± 0.011
1D-CNN	0.449 ± 0.012	0.735 ± 0.038	0.712 ± 0.040	0.720 ± 0.006

#### Testing on CPLM 4.0 independent test dataset with ProtT5 feature

**Figure 2.**
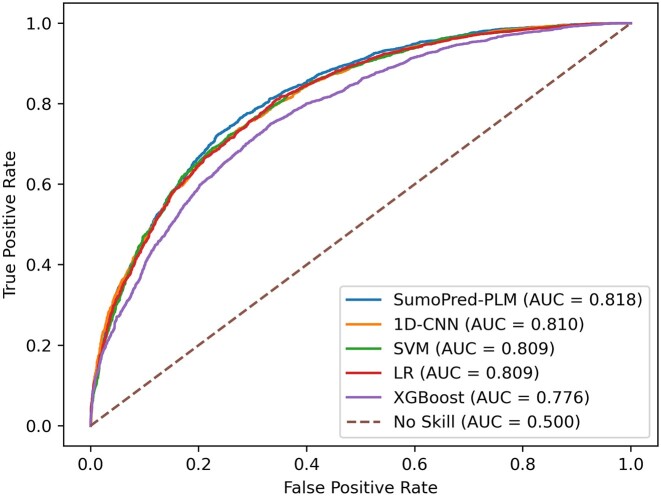
Comparisons of ROC curves of SumoPred-PLM and other models on the SUMOylation CPLM 4.0 independent test dataset. For each model, the area under the ROC curve is reported.

**Figure 3.**
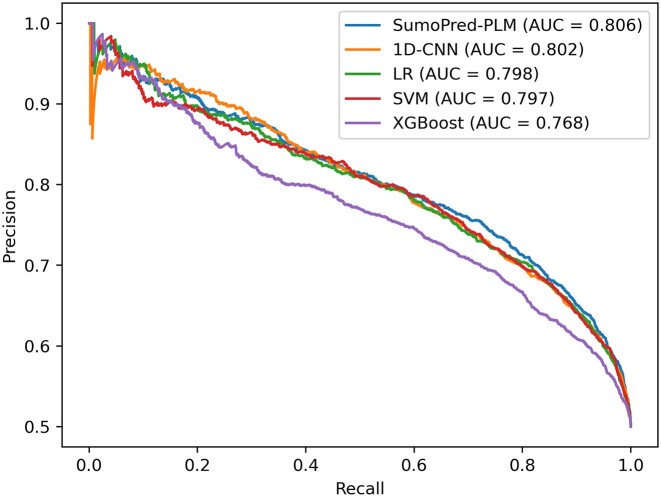
Comparison of precision-recall curves of SumoPred-PLM and other models on the SUMOylation CPLM 4.0 independent test dataset. For each model, the area under the PrAUC is reported.

To assess the performance of our approach on an independent test set with ProtT5 features, we trained the MLP model on the overall CPLM 4.0 training set and evaluated it with CPLM 4.0 SUMOylation independent test data set. It should be noted that none of the positive or negative SUMOylation sites, nor the protein sequences from the CPLM 4.0 independent test set, are present in the CPLM 4.0 training dataset. We rigidly constrained our experiment with this phenomenon because the PLM can learn representations for other sites from the same protein, which can lead to overestimation of the performance. The total number of samples in each set for CPLM 4.0 dataset is shown in Table 1. Our model achieved MCC, SN, SP, and ACC values of 0.4835, 74.02%, 74.32% and 74.17% respectively, on the independent test dataset. Furthermore, MLP was able to classify 2,220 samples as True Negative, 2,212 samples as True Positive, 767 as False Positive and 776 as False Negative. The independent test set result and 10- fold cross-validation results produced by SumoPred-PLM are similar. Moreover, it can be observed from Figure 2 that SumoPred-PLM, which is based on a MLP approach, has the highest area under the receiver operating characteristics curve (ROC). Similarly, Figure 3 shows that SumoPred-PLM has the highest precision-recall area under the curve (PrAUC) compared to other DL and ML approaches. Hence, SumoPred-PLM is a robust computational model for the prediction of SUMOylation PTM in amino acid sequences of proteins. In addition, the SumoPred-PLM MLP model was trained using the dbPTM training dataset (65), utilizing the ProtT5 encoding scheme. Subsequently, the trained model was evaluated with the dbPTM independent test dataset, and the findings are presented in Supplementary Table S4.

Furthermore, McNemar's significant test (78,79) was conducted on the best-performing MLP and SVM classification models. Subsequently, the Chi-square $({\chi ^2})$ distribution value (0.04) was computed and compared with the alpha (0.05) value. Since the ${\chi ^2}$ value is less than the alpha value, we rejected the null hypothesis, suggesting that there is a significant difference between the SVM and MLP classifiers in predicting the outcomes of an independent test dataset. Moreover, the utility of the recently developed ESM2 (3 billion) PLM (80) on the CPLM 4.0 dataset is illustrated in the Supplementary Table S5.

#### Visualization using t-SNE plot

**Figure 4.**
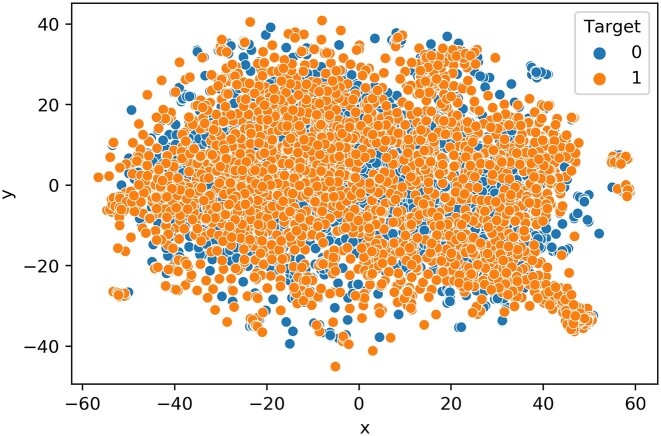
t-SNE illustration of the learned features from ProtT5 language model.

**Figure 5.**
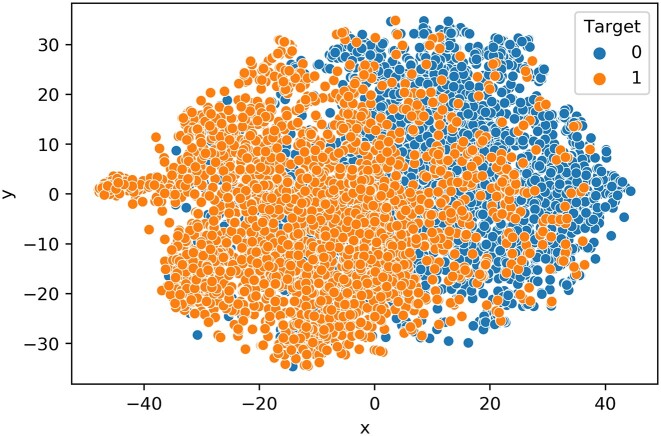
t-SNE illustration of the learned features from the trained MLP model.

Additionally, we investigated the classification efficacy of the features and the learned model using t-SNE visualization technique. Herein, features represent the 1024 numeric vectors of SUMOylated, or non-SUMOylated ‘K’ residues extracted from ProtT5, and the learned model refers to the MLP network trained with the CPLM 4.0 training dataset. To discern the classification effectiveness of these features as well as the feature vector produced by the penultimate hidden layer of the trained MLP network, we used t-SNE to project the features into a two-dimensional space (Figure 4) (81). For the features extracted from ProtT5 on the SUMOylated or non-SUMOylated token ‘K’ of CPLM 4.0 training set, the positive and negative samples are relatively clustered together (Figure 4). Figure 5 represents the t-SNE plot of the feature vectors generated from the penultimate hidden layer of the MLP DL architecture when CPLM 4.0 training set is used. This shows that negative samples (blue points) are concentrated at the right while positive samples (orange points) are concentrated at the left, which indicates that the per residue pre-trained PLM feature extraction with MLP learns SUMOylation patterns and largely clusters positive and negative samples in two-dimensional space. Hence this result demonstrates that contextualized features produced from pretrained ProtT5 when passed to a MLP deep learning network can cluster positive and negative samples of SUMOylation sites in two-dimensional space.

#### 10-fold cross-validation on the CPLM 4.0 training set with Ankh PLM features

In order to scrutinize the usefulness of recent pre-trained PLM Ankh, we performed 10-fold cross-validation on the CPLM 4.0 training dataset with the embeddings from the Ankh PLM (41). The predictive performance of different DL and ML models using the stratified 10-fold cross-validation on the CPLM 4.0 training data set, where the features are extracted from the Ankh PLM is shown in Table 6. The contextualized embeddings (feature vector length = 1536) of the SUMOylated or non-SUMOylated token ‘K’ produced by the pretrained Ankh model when fed to MLP achieves the best performance as seen in Table 6. This MLP model produced MCC, SN, SP and ACC values of 0.464 ± 0.010, 0.752 ± 0.017, 0.711 ± 0.019 and 0.731 ± 0.005 respectively for the stratified 10-fold cross-validation. These large pretrained PLMs have increased capacity to learn and represent complex patterns of proteins, as well as exhibit better performance in terms of accuracy, generalization, and protein language understanding. Moreover, the token capacity (the maximum number of tokens the model can handle during processing), which affects the model's ability to handle long sequences of amino acids, is increased in these large pretrained PLMs. Moreover, the 10-fold cross-validation of explored models on CPLM 4.0 training dataset summarizes that Ankh PLM is shorter than the baseline ProtT5 PLM by slight margins, hence we chose pretrained ProtT5 PLM to encode the protein sequence.

**Table 6.** Results of the 10-fold cross-validation of explored models on the CPLM 4.0 training dataset using Ankh PLM feature encoding. The highest values in each column are highlighted in bold

Models	MCC ± 1 S.D.	SN ± 1 S.D.	SP ± 1 S.D.	ACC ± 1 S.D.
MLP	**0.464 ± 0.010**	**0.752 ± 0.017**	0.711 ± 0.019	**0.731 ± 0.005**
LR	0.421 ± 0.009	0.725 ± 0.004	0.695 ± 0.006	0.710 ± 0.004
XGBoost	0.360 ± 0.010	0.674 ± 0.007	0.685 ± 0.006	0.680 ± 0.005
RF	0.300 ± 0.011	0.573 ± 0.005	0.723 ± 0.006	0.648 ± 0.005
NB	0.194 ± 0.008	0.550 ± 0.005	0.643 ± 0.005	0.596 ± 0.004
SVM	0.461 ± 0.022	0.729 ± 0.017	**0.732 ± 0.015**	0.730 ± 0.011
1D-CNN	0.442 ± 0.010	0.737 ± 0.006	0.704 ± 0.007	0.721 ± 0.005

#### Testing on CPLM 4.0 independent test dataset with Ankh feature

To assess the performance of our approach on an independent test set with Ankh features, we trained the MLP model on the overall CPLM 4.0 training set and appraised the trained model with CPLM 4.0 SUMOylation independent test set. The trained MLP model produced MCC, SN, SP, and ACC of 0.4728, 77.55%, 69.58% and 73.56% respectively, when features from Ankh PLM were used. Furthermore, MLP model trained with Ankh features was able to classify 2077 samples as True Negative, 2315 samples as True Positive, 908 as False Positive, and 670 as False Negative for the CPLM 4.0 independent test dataset. It can be observed from Table 7 that SumoPred-PLM trained with ProtT5 PLM feature representation is better than ANKH PLM feature representation.

**Table 7.** Prediction performance of SumoPred-PLM with ProtT5 and Ankh PLM features on the CPLM 4.0 independent test dataset. The highest values in each column are highlighted in bold

PLM	MCC	SN	SP	ACC
ProtT5	**0.483**	0.740	**0.743**	**0.741**
Ankh	0.472	**0.775**	0.695	0.735

### Performance on the SUMO2/3 dataset

#### 10-fold cross-validation on the SUMO2/3 training set with ProtT5 features

To further examine the robustness of the proposed model, we performed 10-fold cross-validation on the Hendriks *et al.* SUMO2/3 training dataset. The predictive performance of different DL and ML models using the stratified 10-fold cross-validation on the Hendriks *et al.* SUMO2/3 training data set is shown in Table 8. Intriguingly, the same architecture (MLP) produced the highest performance resulting in MCC, SN, SP and ACC values of 0.481 ± 0.017, 0.745 ± 0.029, 0.735 ± 0.027 and 0.740 ± 0.008, respectively for the stratified 10-fold cross-validation. Since the MLP model produced the best result on 10-fold cross-validation, we selected this architecture as our final model and used it to assess the performance on the Hendriks *et al.* SUMO2/3 independent test set.

**Table 8.** Comparison of different learning models on Hendriks *et al.* SUMO2/3 training dataset using 10-fold cross-validation, where features were encoded utilizing ProtT5 PLM. The highest values in each column are highlighted in bold

Models	MCC ± 1 S.D.	SN ± 1 S.D.	SP ± 1 S.D.	ACC ± 1 S.D.
MLP	**0.481 ± 0.017**	**0.745 ± 0.029**	**0.735 ± 0.027**	**0.740 ± 0.008**
LR	0.460 ± 0.020	0.734 ± 0.017	0.725 ± 0.015	0.730 ± 0.010
XGBoost	0.393 ± 0.030	0.695 ± 0.016	0.697 ± 0.018	0.696 ± 0.014
RF	0.356 ± 0.017	0.611 ± 0.010	0.703 ± 0.012	0.676 ± 0.08
NB	0.314 ± 0.015	0.510 ± 0.017	0.791 ± 0.009	0.650 ± 0.008
SVM	0.479 ± 0.014	0.748 ± 0.007	0.730 ± 0.012	0.739 ± 0.007
1D-CNN	0.449 ± 0.019	0.726 ± 0.036	0.721 ± 0.040	0.724 ± 0.009

#### Testing on the SUMO2/3 independent test dataset with ProtT5 feature

To further assess the performance, SumoPred-PLM (MLP model) trained on SUMO2/3 dataset was tested with a SUMO2/3 independent test set. The model produced MCC, SN, SP, ACC of 0.4973, 75.88%, 73.83% and 74.86%, respectively on the SUMO2/3 independent test set. Moreover, the SumoPred-PLM was able to classify 937 samples as True Negative, 963 samples as True Positive, 332 as False Positive, and 306 as False Negative on the SUMO2/3 independent test set.

### Performance on the GPS-SUMO dataset

#### Comparison of SumoPred-PLM with State-of-the-Art predictor on GPS-SUMO dataset

**Figure 6.**
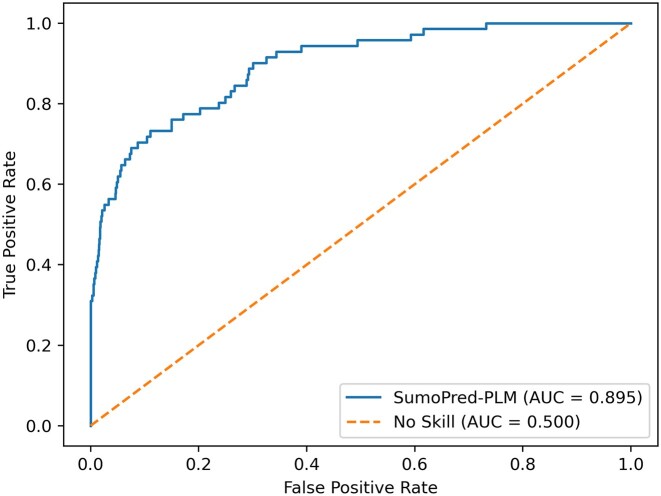
ROC curve of SumoPred-PLM on GPS-SUMO independent test dataset.

To assess the performance of SumoPred-PLM against other approaches, we trained our model on the GPS-SUMO training set and used it to predict SUMOylation sites on the GPS-SUMO independent test set. The MLP model produced MCC, ACC, SN and SP values of 0.3752, 87.56%, 73.23% and 88.30%, respectively on GPS-SUMO independent test dataset. GPS-SUMO produced an area under curve (AUC) of 0.8629 whereas SumoPred-PLM produced an AUC of 0.895 as illustrated in Figure 6. This result is better than the performance of the seminal GPS-SUMO approach, which uses the generation group-based prediction system (GPS) algorithm integrated with Particle Swarm Optimization approach. Furthermore, the MLP classifier was able to classify 1,216 samples as True Negatives, and 52 samples as True Positives. However, it falsely classified 161 samples as False Positive, and 19 samples as False Negative. These results suggest that SumoPred-PLM performs better than the seminal GPS-SUMO method. In addition, it should be noted that SumoPred-PLM was trained and tested with the exact same dataset that was used with the GPS-SUMO approach.

#### Comparison of SumoPred-PLM with SUMOhydro predictor on SUMOhydro dataset

To facilitate a more comprehensive comparison, we have acquired the dataset associated with the SUMOhydro predictor (24). Detailed statistical information pertaining to SUMOhydro dataset is provided in Supplementary Table S6. We extracted the ProtT5 contextualized embedding of the SUMOhydro datasets and then applied these features to the SumoPred-PLM MLP architecture. The results of this analysis are presented in Table 9. It is clear from the results that our predictor outperforms all essential predictors. Additionally, it is important to note that SUMOhydro used a 1:10 ratio of SUMOylation to non-SUMOylation sites in its training dataset. In our experiment, we explored different ratios, and the results indicate that a 1:4 ratio in the training dataset, when combined with ProtT5 embeddings and the MLP architecture, yielded the most optimal outcome. Additionally, SumoPred-PLM MLP model was able to classify 495 samples as True Negative, 17 samples as True Positive, 15 as False Positive, and 7 as False Negative.

**Table 9.** Comparison of SumoPred-PLM with other predictors that were trained with SUMOhydro training dataset and tested with SUMOhydro independent test dataset

Method	SN (%)	SP (%)	ACC (%)	MCC
SumoPred-PLM	**70.8**	**97.0**	**95.8**	**0.592**
JASSA (4)	50.0	94.0	89.3	0.442
SUMOhydro (24)	66.7	93.5	92.3	0.432
SUMOsp2.0 (82)	62.5	92.6	91.2	0.381
seeSUMO-SVM (83)	54.2	95.1	93.3	0.397
seeSUMO-RF (83)	70.8	88.4	87.6	0.351

#### Case studies

**Figure 7.**
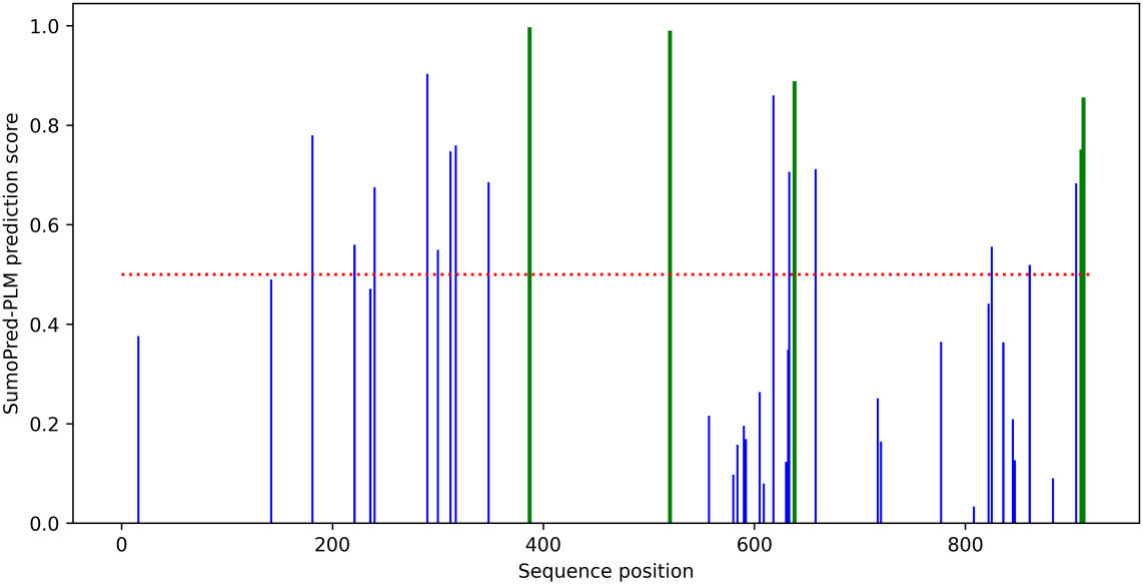
SumoPred-PLM prediction results of human androgen receptor, where sites with a prediction score above 0.5 (shown by the red dotted line) are predicted as SUMOylated sites. Green bars represent the five SUMOylation sites with experimental evidence from protein microarray data.

We performed one case study on the androgen receptor (AR, ANDR_HUMAN UniProt ID: P10275) protein which was not present in training or in the independent test set of CPLM 4.0 dataset. This nuclear steroid receptor is a ligand-activated transcription factor that directs cellular proliferation and differentiation in target tissues (84). Specifically, androgen hormone activated AR binds androgen response elements/ARE on target genes and recruit's coactivator and corepressor proteins to direct gene transcription (85). The AR protein is subject to multiple PTMs including SUMOylation. We and others report AR SUMOylation regulates AR function as our collective whole animal and cell-based studies demonstrate that a disruption of dynamic AR SUMOylation directs aberrant proliferation of prostate and breast cancer cells (86–89). The human androgen receptor protein contains 40 lysine (‘K’) residues. Biochemical studies first identified canonical SUMO consensus sites that include K387 and K520 on AR (highlighted on the Table S3). The K387 and K520 serve as acceptor sites for both SUMO1 and SUMO2/3 modification of endogenous AR protein in several cell lines. However, mono-SUMO1 and poly-SUMO2/3 chains differentially regulate AR function. SUMO1 modification of AR effects transcriptional activity while SUMO2/3 conjugation to AR directs chromatin enrichment and AR protein stability/degradation (90,91). With 40 lysine residues, we postulated that AR protein may exhibit additional non-consensus SUMO motif and possibly even several SUMO paralogue-specific acceptor sites. Our *in silico* analysis with GPS-SUMO of the primary amino acid sequence of AR identified K387 and K520 and three additional SUMO-acceptor sites (K313, K910, K913, Table S3). However, this platform does not distinguish between SUMO paralogue conjugates. Hence, we next evaluated published mass spectrometry data of the endogenous SUMO2/3 proteome from HeLa cells. The dataset reports that 20% of AR is SUMOylated in HeLa cells and eight SUMO2/3-accepting lysine residues of AR are conjugated (five sites analogous to GPS-SUMO and three novel SUMO2/3-acceptors at positions K241, K638, and K823). We then challenged the SumoPred-PLM with the same task of identifying AR SUMOylation sites. As shown in Figure 7, SumoPred-PLM correctly predicts the five validated (K387, K520, K638, K910, K912) SUMO2/3 conjugation sites out of five, thus achieving an accuracy of 100.0% (=5/5). In addition, SumoPred-PLM predicts an additional 14 lysine ‘K’ sites of ANDR_HUMAN protein as positives. Next, we tested if our platform could identify validated and/or novel SUMO2/3 acceptor sites for AR. The model trained with the SUMO2/3 dataset shows 11 SUMO2/3 acceptors at lysine position 181, 290, 387, 520, 618, 638, 658, 861, 905, 910 and 912. Six of the predicted SUMO2/3 sites are novel and previously reported in Hendriks *et al.;* specifically, K181, 290, 618, 658, 861, 905. Hence, these lysine residues that are predicted to be SUMO2/3 modified await experimental validation. Supplementary Table S7 shows prediction results of SumoPred-PLM for all the lysine in ANDR_HUMAN protein.

## Discussion and conclusions

One of the key innovations in SumoPred-PLM is the incorporation of PLM based features to represent protein sequences. PLM based features have proven to be quite useful in various bioinformatics tasks (92–100). Our major goal in the project was to move away from hand-crafted feature extraction for prediction of SUMOylation and SUMO2/3 sites. To achieve this goal, we investigated whether language models learned from a large amount of protein sequences could capture the features predictive of SUMOylation and SUMO2/3 sites. Additionally, we also wanted to investigate what type of machine learning approach would work well on these pre-trained feature representations. Moreover, our other significant contribution is the study of SUMO2/3 data set which was not extensively studied in prior studies. To achieve the goal, we used contextualized embeddings learned from a PLM called ProtT5 to extract features for the site of interest. Subsequently, various ML and DL algorithms were evaluated using 10-fold cross validation and the top performing model was selected as the final model. The MLP model, namely SumoPred-PLM, achieves the best prediction performance among the compared methods as it largely benefits from the knowledge obtained from large sets of protein sequences by the pre-trained ProtT5 model that is used to encode the protein sequences.

SumoPred-PLM does not rely on knowledge of protein structure, nor in the expert-crafted sequence features or time-consuming evolutionary information derived from multiple sequence alignments (MSAs). Instead, the input to the MLP model is a contextual representation of the SUMOylated or non-SUMOylated token ‘K’ from the pre-trained PLM (ProtT5). This state-of-the-art prediction of SUMOylation is likely due to the contextual embeddings of all the amino acids in the protein sequence that are produced by the transformer-based model which makes use of position embedding with a self-attention mechanism. SumoPred-PLM model outperforms the pioneering GPS-SUMO predictor, in the identification of consensus and non-consensus SUMO-acceptor sites. One interesting result portrayed in the t-SNE plot (Figure 5) is that our model was largely able to cluster the two classes of SUMOylated and non-SUMOylated lysine residues in two-dimensional space. SumoPred-PLM is a new approach proposed in this work that uses information distilled from large PLMs to train the DL framework and results an outstanding performance compared to existing approaches. In the future, we will consider using the structural information predicted by AlphaFold2 (101,102) to build models using graph networks (103) for further improving the performance of SUMOylation and SUMO2/3 PTM site prediction.

In addition, we provide a unique service for SumoPred-PLM as a SUMO2/3-specific predictor. To our knowledge, this is the first platform that provides the ability to predict SUMO2/3 paralogue selective acceptor sites. As stated previously, increasing biochemical studies highlight SUMO paralog differentially effect a protein substrate's function and stability. Hence, we anticipate that this SUMO2/3 predictor platform will greatly accelerate the discovery of this SUMO-paralog directed protein effects. For the SUMO2/3 platform, protein machine learning was based on available large-scale SUMO2/3 proteomics data (62). Unfortunately, a similar SUMO1 proteomic analysis is unavailable currently but, when accessible, this dataset can be easily incorporated into the current standing platform.

## Supplementary Material

lqae011_Supplemental_File

## Data Availability

All programs and data are available at https://github.com/PakhrinLab/SumoPred-PLM and https://doi.org/10.6084/m9.figshare.25009160.
