# Supplementary material for: SumoPred-PLM: human SUMOylation and SUMO2/3 sites Prediction using Pre-trained Protein Language Model
Source: NAR Genom Bioinform. 2024 Feb 7;6(1):lqae011. doi: 10.1093/nargab/lqae011 (PMC10849187; doi:10.1093/nargab/lqae011)
Supplement: lqae011_Supplemental_File [file lqae011_supplemental_file.docx]

**SumoPred-PLM: human SUMOylation and SUMO2/3 sites Prediction using Pre-trained Protein Language Model**

Andrew Vargas Palacios^1, †^, Pujan Acharya^1, †^, Anthony Stephen Peidl^2^, Moriah Rene Beck^3^, Eduardo Blanco^4^, Avdesh Mishra^5^, Tasneem Bawa-Khalfe^2^, and Subash Chandra Pakhrin^1, †, *^

^1^ Department of Computer Science and Engineering Technology, University of Houston-Downtown, 1 Main St., Houston, TX 77002, USA

^2^ Department of Biology and Biochemistry, Center for Nuclear Receptors & Cell Signaling, University of Houston, Houston, TX 77204, USA

^3^ Department of Chemistry and Biochemistry, Wichita State University, 1845 Fairmount St., Wichita, KS 67260, USA

^4^ Department of Computer Science, University of Arizona, 1040 4^th^ St., Tucson, AZ 85721, USA

^5^ Department of Electrical Engineering and Computer Science, Texas A&M University-Kingsville, Kingsville, TX 78363, USA

^†^ Contributed Equally

* To whom correspondence should be addressed. Tel: +1-713-221-5819; Fax: +1-713-223-7407; Email: pakhrins@uhd.edu


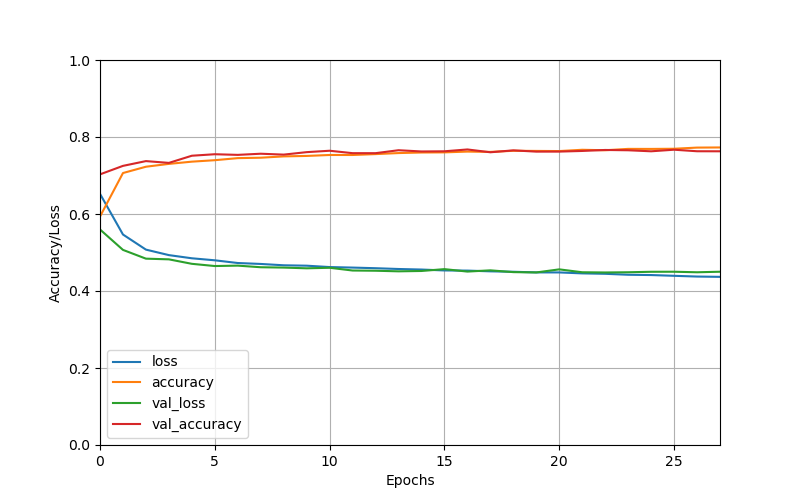


**Figure S1.** Accuracy and loss curve of SumoPred-PLM when trained with CPLM 4.0 SUMOylation datasets.

**dbPTM Dataset**

The dbPTM repository (1) has 1,383 human SUMOylation proteins. To ensure data quality and diversity, we employed psi-cd-hit software (2) to eliminate proteins with a sequence similarity of 30%, resulting in a refined dataset of 1,087 proteins. Subsequently, we partitioned this dataset into two distinct groups: 90% for training (975 proteins) and 10% for independent testing (109 proteins). From these selected proteins, we extracted both the positive and negative SUMOylation sites (“K”) and encoded them using contextualized embeddings obtained from ProtT5 PLM. Table S1 provides a summary of the number of proteins, positive and negative sites in both the training and independent test datasets. We under-sampled the training negative dataset to make it equal to the training positive dataset.

**Table S1.** Positive and negative SUMOylation sites for training and independent testing derived from dbPTM Dataset.

| Dataset | Number of Proteins | Positive | Negative |
| --- | --- | --- | --- |
| Training | 975 | 3,215 | 51,603 |
| Independent Test | 109 | 368 | 368 |

**Table S2.** Hyperparameters of ProtT5 feature-based machine learning models.

| Model name | Hyperparameters |
| --- | --- |
| RF | n_estimators = 100, criterion = entropy |
| XGBoost | max_depth = 3, subsample = 0.8, n_estimators = 200, learning_rate = 0.05, random_state = 5 |
| SVM | C = 0.1, Probability = True, kernel='rbf' |
| NB | GaussianNB() |
| LR | C = 0.01 |
| 1D-CNN | filters = 32, kernel_size = 3, MaxPooling 1D pool_size = 3 |

**Table S3.** Results of the 10-fold cross-validation on the CPLM 4.0 training dataset using different deep learning models encoded with ProtT5 PLM features.

| Models | MCC ± 1 S.D. | SN ± 1 S.D. | SP ± 1 S.D. | ACC ± 1 S.D. |
| --- | --- | --- | --- | --- |
| 1D CNN-BiLSTM | 0.273 ± 0.011 | 0.656 ± 0.035 | 0.616 ± 0.040 | 0.636 ± 0.006 |
| 1D CNN-LSTM | 0.230 ± 0.021 | 0.623 ± 0.054 | 0.605 ± 0.056 | 0.614 ± 0.011 |
| BiLSTM | 0.232 ± 0.016 | 0.630 ± 0.059 | 0.600 ± 0.054 | 0.615 ± 0.008 |
| LSTM | 0.173 ± 0.019 | 0.639 ± 0.045 | 0.532 ± 0.044 | 0.585 ± 0.009 |

**Testing on dbPTM Independent test Dataset with ProtT5 Feature**

We trained the MLP model using a similar architecture as SumoPred-PLM (dbPTM training dataset) and tested it on the unseen independent (dbPTM) test dataset. The trained MLP model produced results as is shown in Table S4. The result produced by the models that were trained by CPLM 4.0, SUMO2/3 and dbPTM human SUMOylation dataset utilizing ProtT5 embedding is similar. Hence this corroborates our experiment results.

**Table S4.** Prediction performance of SumoPred-PLM with ProtT5 features on the dbPTM 4.0 human SUMOylation independent test dataset.

| PLM | MCC | ACC | SN | SP |
| --- | --- | --- | --- | --- |
| ProtT5 | 0.4810 | 0.7404 | 0.7336 | 0.7472 |

**Testing on CPLM 4.0 Independent test Dataset with ESM2 (3 billion) Feature**

We employed the recently developed ESM2 protein language model with 3 billion parameters (ESM2 3B) (3) to encode both the CPLM 4.0 training and independent test datasets. It’s important to highlight that each residue was encoded into feature embeddings of length 2,560. We utilized these ESM2 3B embeddings to train our model and subsequently assessed its performance using the CPLM 4.0 independent test dataset. The outcomes of this evaluation with MLP architecture are presented in Supplementary Table S5. The result clearly indicates that the ProtT5 PLM embeddings are more effective in capturing representative attributes of SUMOylation mechanisms compared to the ESM2 3B PLM embeddings.

**Table S5.** Prediction performance of SumoPred-PLM with ESM2 (3B) features on the CPLM 4.0 human SUMOylation independent test dataset.

| Dataset | MCC | ACC | SN | SP |
| --- | --- | --- | --- | --- |
| dbPTM | 0.4161 | 0.7080 | 0.7063 | 0.7098 |

**SUMOhydro Dataset**

To facilitate a more comprehensive comparison, we have acquired the dataset associated with the SUMOhydro predictor (4). Detailed statistical information pertaining to SUMOhydro dataset is provided in Supplementary Table S6.

**Table S6.** Positive and negative SUMOylation sites for training and independent testing derived from SUMOhydro Dataset.

| Dataset | Number of Proteins | Positive | Negative |
| --- | --- | --- | --- |
| Training | 221 | 358 | 8,071 |
| Independent Test | 24 | 24 | 510 |

**Table S7.** Prediction results of SumoPred-PLM for all the lysine “K” in ANDR_HUMAN protein using CPLM 4.0 trained SUMOylation dataset and Hendriks et al. SUMO2/3 SUMOylation dataset.

|  | SUMO2/3 Prediction | | CPLM 4.0 Prediction | | Remarks |
| --- | --- | --- | --- | --- | --- |
| K-Site | Confidence | Status | Confidence | Status |  |
| 16 | 0.27746788 | FALSE | 0.3760439 | FALSE |  |
| 142 | 0.4020654 | FALSE | 0.48990726 | FALSE |  |
| 181 | 0.54709494 | TRUE | 0.7794659 | TRUE | Predicted SUMO2/3 |
| 221 | 0.34031394 | FALSE | 0.5594331 | TRUE | SUMOylated |
| 236 | 0.44757882 | FALSE | 0.47103974 | FALSE |  |
| 240 | 0.3698572 | FALSE | 0.67510843 | TRUE | SUMOylated |
| 290 | 0.680689 | TRUE | 0.9032195 | TRUE | Predicted SUMO2/3 |
| 300 | 0.41514415 | FALSE | 0.54950047 | TRUE | SUMOylated |
| 312 | 0.45178425 | FALSE | 0.747204 | TRUE | SUMOylated |
| 317 | 0.37371242 | FALSE | 0.7590583 | TRUE | SUMOylated |
| 348 | 0.46500766 | FALSE | 0.6852754 | TRUE | SUMOylated |
| **387** | 0.98777235 | TRUE | 0.99477226 | TRUE | Predicted SUMO2/3 |
| **520** | 0.98876965 | TRUE | 0.9880437 | TRUE | Predicted SUMO2/3 |
| 557 | 0.22396764 | FALSE | 0.21585198 | FALSE |  |
| 580 | 0.40916798 | FALSE | 0.09761692 | FALSE |  |
| 584 | 0.3249809 | FALSE | 0.15734835 | FALSE |  |
| 590 | 0.18790391 | FALSE | 0.19552702 | FALSE |  |
| 592 | 0.4149146 | FALSE | 0.16889413 | FALSE |  |
| 605 | 0.20873798 | FALSE | 0.2636523 | FALSE |  |
| 609 | 0.17193241 | FALSE | 0.07959153 | FALSE |  |
| 618 | 0.5542835 | TRUE | 0.859822 | TRUE | Predicted SUMO2/3 |
| 630 | 0.363217 | FALSE | 0.12329262 | FALSE |  |
| 632 | 0.25119996 | FALSE | 0.3488827 | FALSE |  |
| 633 | 0.46320546 | FALSE | 0.7062703 | TRUE | SUMOylated |
| 638 | 0.9485618 | TRUE | 0.88646185 | TRUE | Predicted SUMO2/3 |
| 658 | 0.73875433 | TRUE | 0.71149015 | TRUE | Predicted SUMO2/3 |
| 717 | 0.2933737 | FALSE | 0.25086835 | FALSE |  |
| 720 | 0.3499453 | FALSE | 0.16383304 | FALSE |  |
| 777 | 0.14014974 | FALSE | 0.36442462 | FALSE |  |
| 808 | 0.06947713 | FALSE | 0.03339902 | FALSE |  |
| 822 | 0.24394864 | FALSE | 0.44081077 | FALSE |  |
| 825 | 0.4099988 | FALSE | 0.5557054 | TRUE | SUMOylated |
| 836 | 0.25226128 | FALSE | 0.36368915 | FALSE |  |
| 845 | 0.07059266 | FALSE | 0.20896572 | FALSE |  |
| 847 | 0.23488313 | FALSE | 0.12637271 | FALSE |  |
| 861 | 0.5095632 | TRUE | 0.51911455 | TRUE | Predicted SUMO2/3 |
| 883 | 0.16107537 | FALSE | 0.08986624 | FALSE |  |
| 905 | 0.5757346 | TRUE | 0.68318814 | TRUE | Predicted SUMO2/3 |
| 910 | 0.636209 | TRUE | 0.74928164 | TRUE | Predicted SUMO2/3 |
| 912 | 0.93208236 | TRUE | 0.8538785 | TRUE | Predicted SUMO2/3 |

**REFERENCES**

1. Li, Z., Li, S., Luo, M., Jhong, J.-H., Li, W., Yao, L., Pang, Y., Wang, Z., Wang, R., Ma, R. *et al.* (2021) dbPTM in 2022: an updated database for exploring regulatory networks and functional associations of protein post-translational modifications. *Nucleic Acids Research*, **50**, D471-D479.

2. Huang, Y., Niu, B., Gao, Y., Fu, L. and Li, W. (2010) CD-HIT Suite: a web server for clustering and comparing biological sequences. *Bioinformatics*, **26**, 680-682.

3. Lin, Z., Akin, H., Rao, R., Hie, B., Zhu, Z., Lu, W., Smetanin, N., Verkuil, R., Kabeli, O., Shmueli, Y. *et al.* (2023) Evolutionary-scale prediction of atomic-level protein structure with a language model. *Science*, **379**, 1123-1130.

4. Chen, Y.Z., Chen, Z., Gong, Y.A. and Ying, G. (2012) SUMOhydro: a novel method for the prediction of sumoylation sites based on hydrophobic properties. *PLoS One*, **7**, e39195.
